# Breast density and estradiol are associated with distinct different expression patterns of metabolic proteins in normal human breast tissue *in vivo*

**DOI:** 10.3389/fonc.2023.1128318

**Published:** 2023-03-29

**Authors:** Jimmy Ekstrand, Annelie Abrahamsson, Peter Lundberg, Charlotta Dabrosin

**Affiliations:** ^1^ Department of Oncology and Department of Biomedical and Clinical Sciences, Linköping University, Linköping, Sweden; ^2^ Department of Radiation Physics and Department of Medical and Health Sciences, Linköping University, Linköping, Sweden; ^3^ Center for Medical Image Science and Visualization (CMIV), Linköping University, Linköping, Sweden

**Keywords:** mammography, microdialysis, sex steroids, estradiol, breast density

## Abstract

**Background:**

Breast density and exposure to sex steroids are major risk factors for breast cancer. The local microenvironment plays an essential role in progression of breast cancer. Metabolic adaption is a major hallmark of cancer. Whether proteins from the extracellular space regulating metabolism are affected in breast cancer, dense breasts or by estrogen exposure are not yet fully elucidated.

**Methods:**

Women with breast cancer, postmenopausal women with normal breast tissue with varying breast density or premenopausal women with breasts exposed to high levels of estradiol were included in the study. Microdialysis was used to collect proteins from the extracellular space *in vivo* in 73 women; 12 with breast cancer, 42 healthy postmenopausal women with different breast densities, and 19 healthy premenopausal women. Breast density was determined as lean tissue fraction (LTF) using magnetic resonance imaging. Data were evaluated in a murine breast cancer model. We quantified a panel of 92 key proteins regulating metabolism using proximity extension assay.

**Results:**

We report that 29 proteins were upregulated in human breast cancer. In dense breasts 37 proteins were upregulated and 17 of these were similarly regulated as in breast cancer. 32 proteins correlated with LTF. In premenopausal breasts 19 proteins were up-regulated and 9 down-regulated. Of these, 27 correlated to estradiol, a result that was confirmed for most proteins in experimental breast cancer. Only two proteins, pro-cathepsin H and galanin peptide, were similarly regulated in breast cancer, dense- and estrogen exposed breasts.

**Conclusions:**

Metabolic proteins may be targetable for breast cancer prevention. Depending on risk factor, this may, however, require different approaches as breast density and estradiol induce distinct different expression patterns in the breast. Additionally, metabolic proteins from the extracellular space may indeed be further explored as therapeutic targets for breast cancer treatment.

## 1 Introduction

Two major independent risk factors for breast cancer are mammographic dense breast tissue and exposure to sex steroids (1, 2). To date, the biological mechanisms that govern these processes remain elusive.

There is a 4-6-fold increased risk of breast cancer for women with dense breast as compared to women with nondense breasts and an inverse relationship with nondense area and risk of the disease has been shown (2). Women with > 50% dense area account for approximately 30% of all breast cancer cases (2). Dense breast tissue is characterized by high amounts of stroma, including collagen, in contrast to nondense breasts where fat is the major component (3). The proportion of breast epithelial cells in normal breast tissue is less than 10% and there are no conclusive data on differences on the quantity or proliferation rate of these cells depending on breast density (3–5).

Another major independent risk factor for breast cancer is exposure to sex steroids including estrogens (1). No association between circulating estrogen levels and breast density has been observed (2). Sex steroids, including estrogens, play a critical role in the regulation of the epithelial cell proliferation and apoptosis in normal breast tissue (6). However, only a minor fraction of the epithelial cells proliferates during the menstrual cycle (6). Additionally, the response in epithelial cells is highly dependent upon epithelial-stroma interactions in the microenvironment, which also are affected by estrogens (7). Estradiol has profound effects on the local immune microenvironment, angiogenesis, and fibroblasts function in the breast (8–12). Thus, it is rather the microenvironment surrounding the epithelial cells than the epithelial cells alone that determines the risk of breast cancer initiation and progression as an activated stroma is a prerequisite for tumor formation.

Metabolic reprogramming is included in the hallmarks of cancer (13). During progression cancer acquires various metabolic phenotypes in cooperation with stromal cells (14). These metabolic properties may enable cancer cells in dormant tumors to increase cell survival, which supports hyperplastic growth, increase invasion capacities, and evade immune surveillance (15). The metabolic phenotype in a tissue or in cancer is a result of complex interactions of intrinsic processes in epithelial or cancer cells and extrinsic factors in the microenvironment (14). The importance of cancer metabolism is supported by recent data suggesting that therapeutic targets in the microenvironment, including metabolism, may be more important than oncogenes (16).

To date it is unclear whether normal tissues with intrinsically increased risk of cancer express altered metabolic properties that support tumor initiation and early cancer progression. Whether proteins involved in metabolic pathways are affected in normal human breasts by two major risk factors for breast cancer, breast density and estradiol exposure, are previously not explored. Additionally, whether proteins sampled from the extracellular space involved in metabolism are altered in human breast cancer *in vivo* is previously not determined. Here we used microdialysis to sample proteins directly from live breast tissue. The advantage of this approach is that proteins can be quantified directly in the target organ, albeit its invasiveness, as compared to blood levels that will reflect circulating levels originating from many different organs in the body.

By using a panel of 92 key proteins involved in metabolism we explored whether levels were altered in the extracellular local microenvironment *in vivo* in breast cancer compared to normal breast tissue, in normal breast tissue depending on breast density or in breasts exposed to high levels of estradiol compared to breasts with low estradiol levels.

## 2 Materials and methods

### 2.1 Subjects

Previously collected and biobanked samples from different cohorts were used in the exploratory clinical study. The Regional Ethical Review Board of Linköping approved the collections which were carried out in accordance with the Declaration of Helsinki. All subjects gave informed consent. A total of 73 women were included.

Twelve postmenopausal women (ages 52-86 years) with breast cancer were investigated with microdialysis before surgery. All breast cancers were estrogen receptor (ER) positive and human epidermal growth factor 2 (HER-2) negative.

Healthy postmenopausal women from the mammography screening program at Linköping University Hospital that were categorized according to the Breast Imaging Reporting and Data System (BI-RADS) as either entirely fatty nondense (BI-RADS A) or extremely dense (BI-RADS D) were invited to the study (17). Forty-two healthy postmenopausal women (ages 55–74 years) were consecutively recruited for the study. The women were subjected to magnetic resonance imaging (MRI) (18, 19). On the MRI lean tissue fraction (LTF), as a continuous measure of breast density, was calculated in a volume selection of 20 x 20 x 20 mm in the upper lateral quadrant of the left breast, as previously described (19, 20). In brief, 1.5 T Achieva MR scanner (Philips Healthcare, Best, Netherlands) using a dual breast seven-element breast coil was used. Water- and fat separated MR images were computed, as previously described (21), in summary: axial 3D 6-echo turbo field echo MRI images, anterior-posterior frequency encoding, first TE at 2.3 ms and ΔTE of 2.3 ms, TR 15.4 ms, flip angle 10°, 300×300×150 mm^3^ field of view, 200×200 scan matrix and 3 mm slice thickness. LTF was computed as the ratio of lean tissue volume to total volume.

After a second review of the mammograms, it was noticed that two women had been miscategorized and intermediate with respect to breast density. These two women were not included in the analyses of dense vs. nondense breast but included in the correlation analyses.

For the premenopausal group, 19 nulliparous women (ages 20–32 years) with a history of regular menstrual cycles (cycle length, 27–34 days) were included. All of these were investigated with microdialysis in the luteal phase of the menstrual cycle.

None of the healthy volunteer women had a history of breast cancer or were currently using (or had used within the past 3 months) hormone replacement therapy, sex steroid-containing contraceptives, anti-estrogen therapies, including selective estrogen receptor modulators, or degraders.

### 2.2 Microdialysis procedure

Prior to insertion of the microdialysis catheters 0.5 mL lidocaine (10 mg*/*mL) was administrated intracutaneously. Microdialysis catheters (M Dialysis AB, Stockholm, Sweden), which consisted of a tubular dialysis membrane (diameter 0.52 mm, 100,000 atomic mass cut-off) glued to the end of a double-lumen tube were inserted *via* a splittable introducer (M Dialysis AB), connected to a microinfusion pump (M Dialysis AB) and perfused with 154 mmol*/*L NaCl and 60 g/L hydroxyethyl starch (Voluven^®^; Fresenius Kabi, Uppsala, Sweden), at 0.5 µL/min. The women with ongoing breast cancer were investigated with 10 mm long membranes; one catheter was inserted within the cancer tissue and the other into normal adjacent breast tissue. The healthy volunteer women were investigated with 20 mm long microdialysis membranes; one was placed in the upper lateral quadrant of the left breast and directed towards the nipple as previously described (9, 22–29). The premenopausal women were subjected to microdialysis in the luteal phase of the menstrual cycle. The microdialysis catheter was placed in the same quadrant where LTF was determined.

After a 60 min equilibration period, the outgoing perfusate was stored at -80°C for subsequent analysis.

### 2.3 Breast cancer model

The Institutional Animal Ethics Committee at Linköping University approved this study, which conformed to regulatory standards of animal care. Oophorectomized athymic mice (Balb/C-nu/nu, 6-8 weeks old, Scanbur, Sweden) were housed at Linköping University in ventilated cages with a light/dark cycle of 12/12 hours with rodent chow and water available *ad libitum*. Mice were anesthetized *via* intraperitoneal (i.p.) injection of ketamine/xylazine and implanted with a s.c. 3 mm pellet containing either 17β-estradiol (0.18 mg/60-day release, Innovative Research of America, Sarasota, FL, USA) or placebo. The active pellet releases serum concentrations of 150-250 pM estradiol. 5 × 10^6^ MCF-7 cells were injected into the dorsal mammary fat pads in 200 µL PBS. MCF-7 cells require estrogen for tumor formation and growth in mice, therefore, a non-estrogen control group is not possible to achieve. When tumors reached ≈20 mm^2^ in size the mice were treated with fulvestrant (5 mg/mouse twice per week, s.c.) in addition to the estradiol exposure.

### 2.4 Microdialysis in mice

Tumor-bearing mice with size-matched tumors were anesthetized with i.p. injections of ketamine/xylazine and maintained by repeated s.c. injections of ketamine/xylazine. Body temperatures were maintained using a heat lamp. Microdialysis probes with 4-mm membranes (CMA 20, 100 kDa cutoff; CMA Microdialysis AB, Kista, Sweden) were inserted into tumor tissue and connected to a microdialysis pump (CMA 102; CMA Microdialysis AB) perfused at 0.6 μl/min with 154 mmol*/*L NaCl and 60g/L hydroxyethyl starch (Voluven^®^; Fresenius Kabi, Uppsala, Sweden), as previously described (30, 31). After a 60 min equilibrium period, outgoing perfusates (*i.e*., microdialysates) were collected and stored at -80°C for subsequent analysis.

### 2.5 Protein quantifications

The Metabolism panel (Olink Bioscience, Uppsala Sweden) was used for the microdialysis samples. Proteins included in the panel are listed in **Supplementary Table 1**. Although some of these proteins are considered to be of cellular origin, only one protein, ANXA4, was undetectable in human plasma during the development of the assay (Olink Bioscience, Uppsala Sweden). Thus 91 out of the 92 proteins were quantified circulating in plasma of healthy individuals during the validation of the assay; https://www.olink.com/content/uploads/2021/09/olink-metabolism-validation-data-v2.0.pdf. In the present study, microdialysis samples were analyzed with multiplex proximity extension assay (PEA, Olink Bioscience, Uppsala Sweden) as previously described (32–34). In brief, 1 μL microdialysis sample was incubated with proximity antibody pairs tagged with DNA reporter molecules. The DNA tails formed an amplicon by proximity extension, which was quantified by high-throughput real-time PCR (BioMark™ HD System; Fluidigm Corporation, South San Francisco, CA, USA). The generated fluorescent signal correlates with protein abundance by quantitation cycles (Cq) produced by the BioMark Real-Time PCR Software. To minimize variation within and between runs, the data were normalized using both an internal control (extension control) and an interplate control and transformed using a predetermined correction factor. The pre-processed data were provided in the arbitrary unit normalized protein expression (NPX) on a log_2_ scale, which were then linearized by using the formula 2^NPX^. A high NPX value corresponds to high protein concentrations. Values represented a relative quantification meaning that no comparison of absolute levels between different proteins could be made.

Protein-protein interactions were analyzed using the STRING data base.

### 2.6 Estradiol analysis

Estradiol levels were analyzed using a high sensitivity immunoassay kit (DRG International, Springfield Township, NJ, USA).

### 2.7 Statistical analyses

Statistical analyses were performed using nonparametric Wilcoxon matched-pairs signed rank tests or Kruskal Wallis tests followed by unpaired Mann-Whitney U tests when more than two groups were compared as the data was non-normally distributed. Spearman’s correlation test was used for calculations of correlations. A *P*<0.05 was considered statistically significant. Statistics were performed with Prism 9.0 (GraphPad, San Diego, CA, USA).

## 3 Results

There were no statistically significant differences in BMI, age or local breast estradiol levels between dense and nondense group; BMI (mean ± SD) 24 ± 3.3 vs 25 ± 3.2, age (years, mean ± SD) 64 ± 5.8 vs 65 ± 5.2 respectively and local breast estradiol (pmol/L mean ± SD) 40 ± 14 vs 40 ± 12 respectively.

In the premenopausal group BMI (mean ± SD) was 24 ± 1.5 and local breast estradiol levels (pmol/L mean ± SD) were 196 ± 40.

### 3.1 Distinct patterns of proteins in breast cancer, postmenopausal dense- and premenopausal breasts

**Figure 1:**
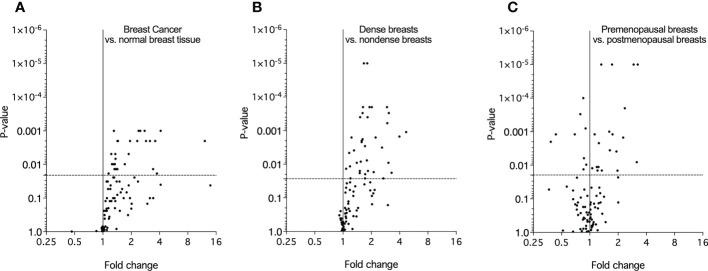
Molecular characterization of the extracellular microenvironment *in vivo* in breast cancer, postmenopausal dense breasts vs. nondense breasts, and in premenopausal breasts vs postmenopausal dense breasts. Microdialysis was performed for *in vivo* collection of proteins from the extracellular space that were quantified as described in the Materials and Methods. Volcano plots illustrate the log_10_ statistical significance (FDR-adjusted p-value) in relation to the log_2_ fold change of 92 proteins involved in metabolism. **(A)**. Twelve patients with breast cancer underwent microdialysis one day prior to their surgery. One catheter was inserted into the breast cancer and another catheter was inserted into normal adjacent breast tissue. Fold change of proteins in cancer tissue as compared to normal adjacent breast tissue. The dotted line indicates the FDR-adjusted *p*-value, <0.021. **(B)**. 40 healthy postmenopausal women with dense breast tissue (n=20) or nondense breast tissue (n=20) were subjected to microdialysis in the upper lateral quadrant of the left breast. Fold change was calculated from the median value of proteins in dense vs. nondense breasts. The dotted line indicates the FDR-adjusted *p*-value, <0.027. **(C)**. 19 premenopausal women were subjected to microdialysis in the upper lateral quadrant of the left breast in the luteal phase of the menstrual cycle. Fold change was calculated from median values of proteins in premenopausal breast vs. postmenopausal dense breasts (n=20). The dotted line indicates the FDR-adjusted *p*-value, <0.02.

**Figure 2:**
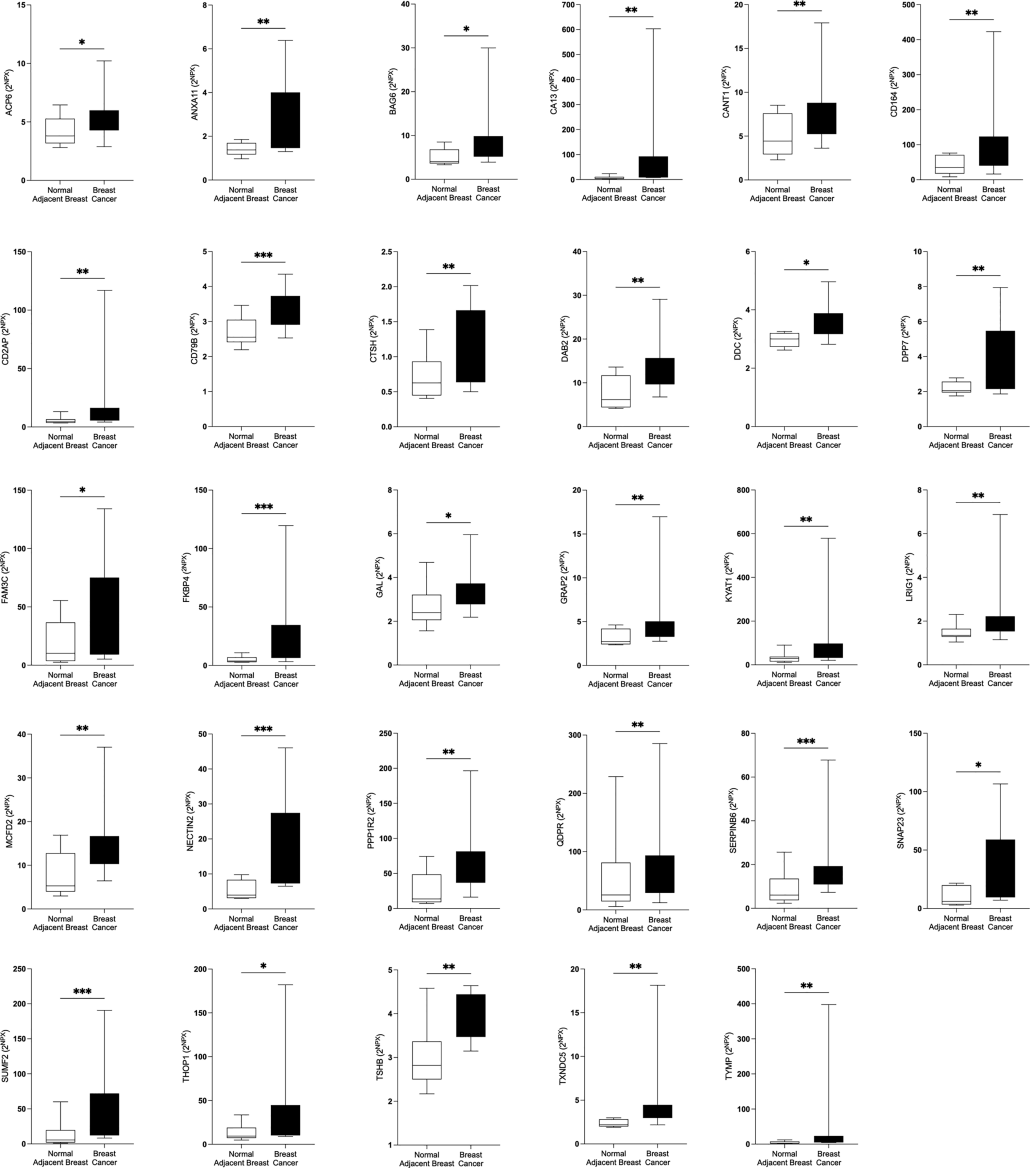
Significantly altered extracellular levels of proteins regulating metabolism in human estrogen receptor positive (ER+) breast cancer *in vivo*. Twelve patients with breast cancer underwent microdialysis one day prior to their surgery. One catheter was inserted into the breast cancer and another catheter was inserted into normal adjacent breast tissue for *in vivo* collection of proteins from the extracellular space. The levels of the significantly altered proteins that were depicted in **Figure 1A** are shown. Data are presented as box plots with whiskers of min and max values. *P<0.05, **P<0.01, ***P<0.001.

**Figure 3:**
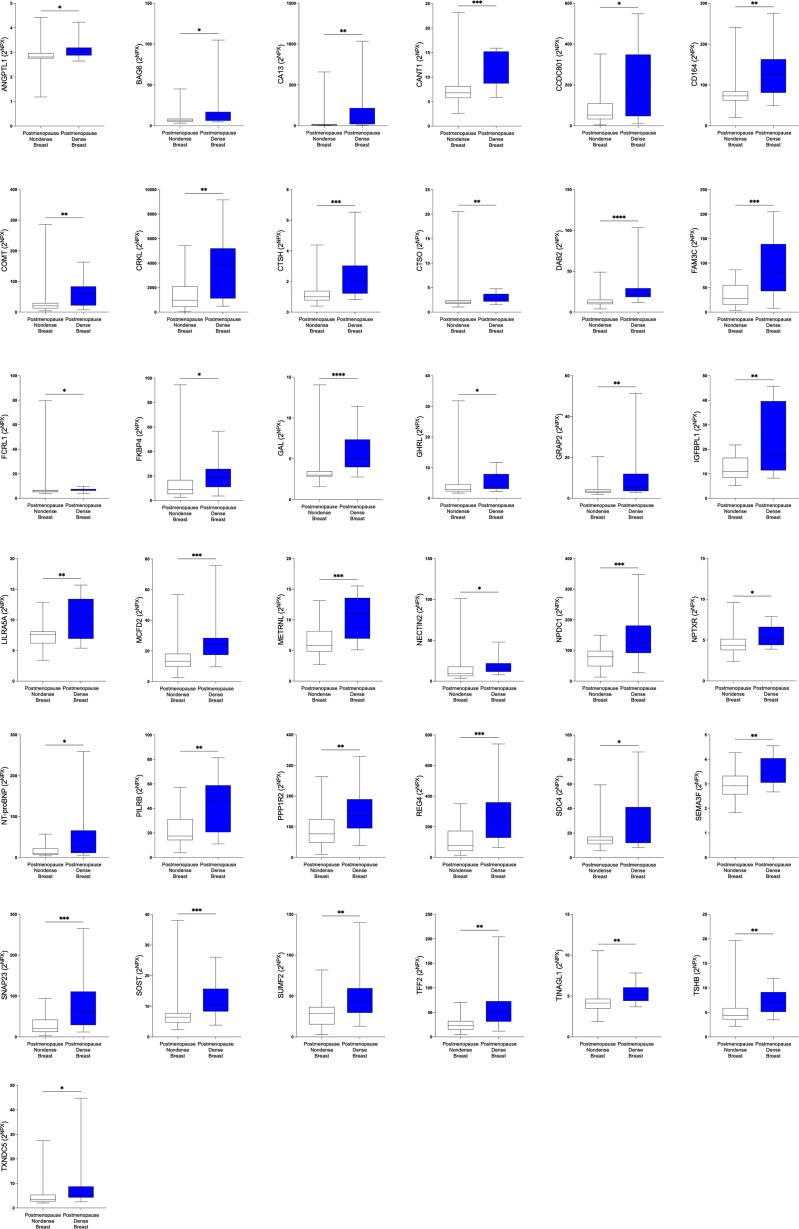
Significantly altered extracellular levels of proteins regulating metabolism in postmenopausal dense and nondense breast tissue. 40 healthy postmenopausal women with dense breast tissue (n=20) or nondense breast tissue (n=20) were subjected to microdialysis in the upper lateral quadrant of the left breast for *in vivo* collection of proteins from the extracellular space. The levels of the significantly altered proteins that were depicted in **Figure 1B** are shown. Data are presented as box plots with whiskers of min and max values. *P<0.05, **P<0.01, ***P<0.001.

**Figure 4:**
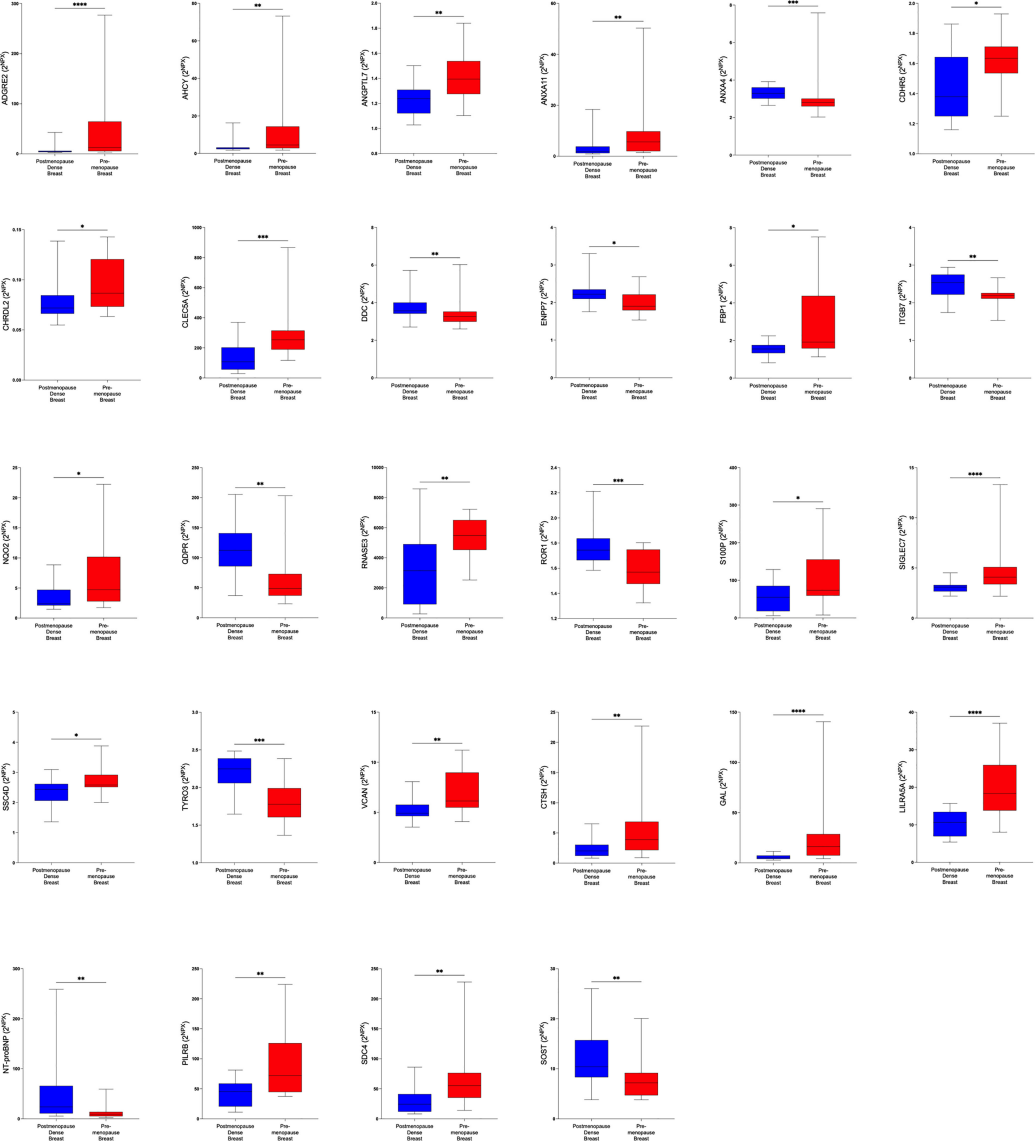
Significantly altered extracellular levels of proteins regulating metabolism in premenopausal breasts and postmenopausal dense breasts. 19 healthy premenopausal women in the luteal phase of the menstrual cycle and 20 healthy postmenopausal women with dense breast tissue were subjected to microdialysis in the upper lateral quadrant of the left breast. The levels of the significantly altered proteins that were depicted in **Figure 1C** are shown. Data are presented as box plots with whiskers of min and max values.*P<0.05, **P<0.01, ***P<0.001.

In the first set of analyses, we determined whether any of the proteins were significantly altered in breast cancer *in vivo* as a measure of the biological relevance for human disease. As shown in **Figure 1A**, 29 proteins, out of the panel of 92, were significantly up-regulated after FDR correction in breast cancers as compared to normal adjacent breast tissue. Thereafter we investigated whether dense breast tissue without any pathology as compared to normal nondense breasts in postmenopausal women exhibited any alterations of the 92 proteins. As shown in **Figure 1B**, 37 proteins were up-regulated in dense breasts. In premenopausal breast tissue, which per definition is dense, 28 proteins were significantly changed compared to postmenopausal dense breast, 19 were up-regulated and 9 down-regulated, **Figure 1C**. In **Figure 2** all individual proteins that were changed in breast cancer are depicted. In **Figures 3**, **4** altered proteins in dense breasts and premenopausal breasts respectively are shown.

Protein-protein interactions are shown in Supplementary **Figures 1A-C**.

### 3.2 Similar patterns of altered proteins in dense breast as in breast cancer

**Figure 5:**
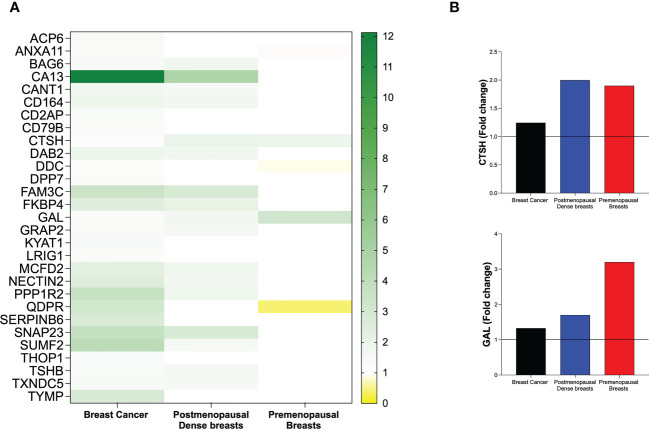
Heat map of extracellular *in vivo* metabolic proteins that were identified as significantly altered in human breast cancer. Microdialysis was performed for *in vivo* collection of proteins from the extracellular space that were quantified in as described in the Materials and Methods in three cohorts of women; 12 with ER+ breast cancer, 40 postmenopausal healthy women with dense (n=20) or nondense (n=20) breast tissue, and 19 premenopausal women investigated in the luteal phase of the menstrual cycle. **(A)** Left column depicts all 29 proteins that were identified as up-regulated in human estrogen receptor positive breast cancer patients as compared to normal adjacent breast tissue. Mid column depicts the regulation of the proteins in postmenopausal dense breast tissue vs. postmenopausal nondense breast. Right column depicts the regulation of the proteins in premenopausal breast tissue vs. postmenopausal dense breast tissue. **(B)** Fold change of the only two proteins that were similarly regulated in the three cohorts: Pro-cathepsin H (CTSH) and Galanin peptides (GAL).

Of the 29 significantly up-regulated proteins in breast cancer, 17 were also up-regulated in postmenopausal dense breasts as compared to postmenopausal nondense breasts. However, in premenopausal breast, who have by definition dense breasts, as compared to postmenopausal dense breast three of these proteins were up-regulated whereas two were down-regulated, **Figure 5A**. Only two proteins exhibited similar alteration in the three different cohorts; Pro-cathepsin H (CTSH) and Galanin peptides (GAL), which were up-regulated in all groups, **Figure 5B**.

### 3.3 Distinct different patterns of expression levels of protein in dense breast and premenopausal breast

**Figure 6:**
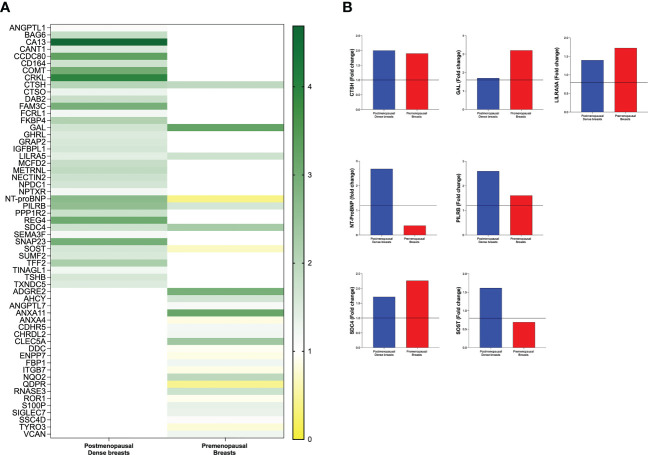
Distinct different patterns of expression levels of protein in dense breasts and premenopausal breasts. Microdialysis was performed for *in vivo* collection of proteins from the extracellular space that were quantified in as described in the Materials and Methods in 40 postmenopausal healthy women with dense (n=20) or nondense (n=20) breast tissue, and 19 premenopausal women investigated in the luteal phase of the menstrual cycle. **(A)** Left column depicts 37 proteins that were identified to be significantly altered in postmenopausal dense breast tissue vs. postmenopausal nondense breast and right column depicts the 28 proteins that were identified to be significantly altered in premenopausal breast tissue vs. postmenopausal dense breast tissue. **(B)** The seven that were significantly altered in both postmenopausal dense breasts vs. nondense breasts and in premenopausal breasts vs. postmenopausal dense breasts. Five proteins were up-regulated in both cohorts whereas two proteins were up-regulated in dense breasts and down-regulated in premenopausal breasts.

In the next analysis we compared which proteins that were up-regulated in postmenopausal dense breast tissue as compared to postmenopausal nondense breasts. As shown in **Figure 6A** 37 proteins were, after FDR correction, significantly up-regulated in dense breast. In premenopausal breast 28 proteins were significantly altered; 19 were up-regulated and 9 significantly down-regulated, **Figure 6A**. Seven proteins were shared between the groups; CTSH, GAL, leukocyte immunoglobulin-like receptor subfamily A member 5 (LILRA5), paired immunoglobulin-like type 2 receptor beta (PILRB), and syndecan-4 (SDC4), which were up-regulated in both tissues. Two proteins were up-regulated in dense breast but down-regulated in premenopausal breasts: N-terminal prohormone of brain natriuretic peptide (NT-ProBNP) and sclerostin (SOST), **Figure 6B**.

### 3.4 Correlations with breast density and estradiol

**Figure 7:**
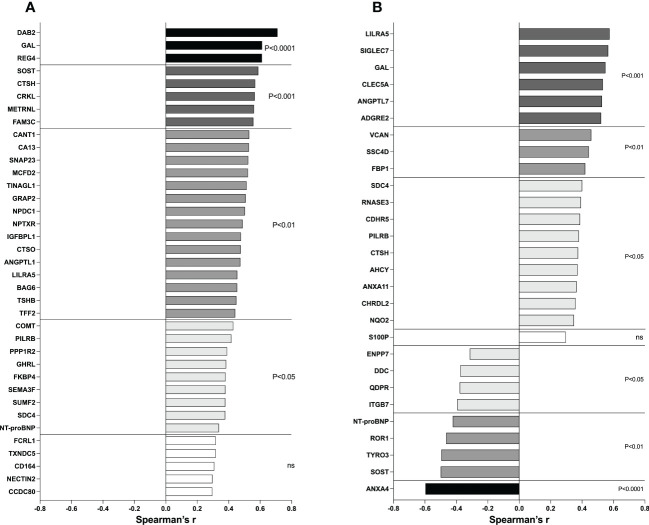
Correlations between metabolic proteins and LTF and estradiol. **(A)** Postmenopausal women underwent MRI and microdialysis for collection of proteins from the extracellular space as described in the Materials and Methods. Correlation analysis between proteins and breast density (LTF) was performed. **(B)** Postmenopausal women with dense breast tissue and premenopausal women underwent microdialysis as described in the Materials and Methods. Correlation analysis between proteins and local breast estradiol was performed. Bars represent Spearman’s rank correlation coefficients. Colored bars indicate statistical significance, and white bars indicate ns=not significant.

Thereafter we wanted to investigate whether breast density and estradiol correlated with the proteins that were significantly altered in the breasts as this would strengthen an involvement of these two measures on the regulation of proteins in the extracellular space. For breast density, we used the precise continuous measure of density calculated from MRI namely LTF, and for estradiol we used the local breast tissue levels. As shown in **Figure 7A**, 32 out of the 37 proteins that were significantly altered in dense breast also correlated significantly with LTF supporting a role of breast density in the regulation of these proteins. In **Figure 7B**, 27 out of 28 proteins that were significantly up- or down-regulated in premenopausal breast also exhibited significant correlations with estradiol levels in the breast.

As CTSH has been suggested to regulate GAL we tested if these two proteins correlated in our data set. Indeed, a strong positive correlation was found, Spearman’s r=0.857, p<0.00001.

### 3.5 An E2 dependent regulation of proteins was corroborated in experimental breast cancer

Next, we wanted to explore whether the proteins that were associated with local estradiol levels in normal human breast tissue were estrogen regulated in experimental ER+ breast cancer in mice. 18 of the 27 proteins that correlated with estradiol in normal human breast tissue were quantifiable in the murine microdialysis samples. The following proteins were below the lowest level of detection in all samples; sialic acid-binding Ig-like lectin 7 (SIGLEC7), c-type lectin domain family 5 member A (CLEC5A), eosinophil cationic protein (RNASE3), paired immunoglobulin-like type 2 receptor beta (PILRB), chordin-like protein 2 (CHRDL2), ribosyldihydronicotinamide dehydrogenase (NQO2), NT-proBNP, inactive tyrosine-protein kinase transmembrane receptor ROR1 (ROR1), SOST, annexin A4 (ANXA4) and thus, could not be analyzed for estrogen dependency.

**Figure 8:**
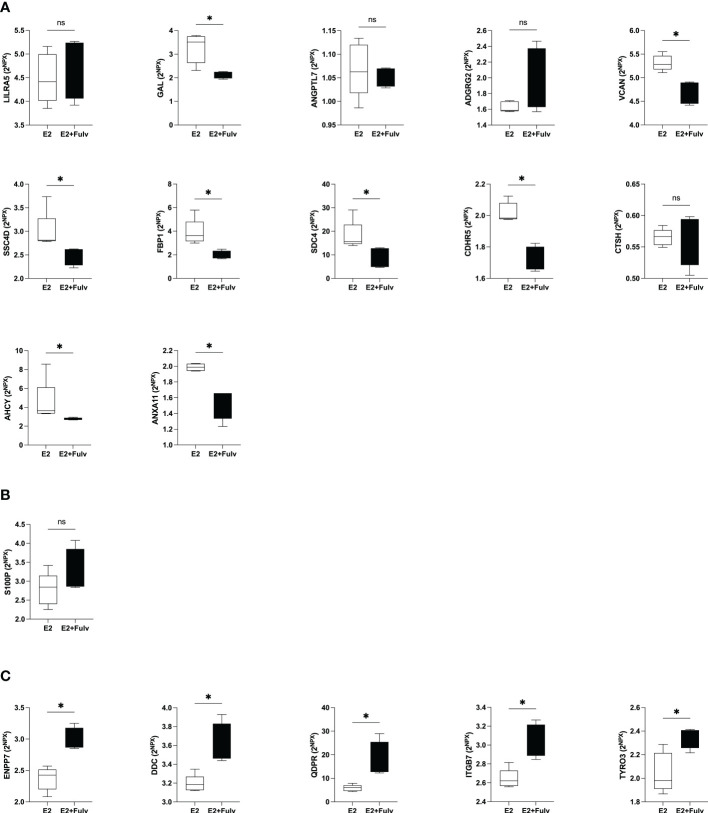
Hormonal regulation of extracellular metabolic proteins in estrogen receptor (ER+) experimental breast cancer. Oophorectomized athymic mice supplemented with physiological levels of estradiol (E2) were injected with MCF-7 cells into the dorsal mammary fat pads. At similar tumor sizes, mice either continued with E2 or were additionally treated with fulvestrant (E2+Fulv) (5 mg/mouse every 3 days, s.c.). Size-matched tumors from the different treatment groups underwent microdialysis for sampling of proteins from the extracellular space *in vivo*. **(A)** Proteins that were significantly positively correlated with estradiol in human breast tissue. **(B)** Protein that was up-regulated in premenopausal breast tissue but failed to show any correlation with local breast E2 levels. **(C)** Proteins that were significantly negatively correlated with estradiol in human tissue. *P<0.05, ns= not significant.

As shown in **Figure 8A**, eight of the proteins that were significantly positive correlated with estradiol were downregulated by the anti-estrogen fulvestrant therapy in mice; GAL, versican core protein (VCAN), scavenger receptor cysteine-rich domain-containing group B protein (SSC4D), fructose-1,6-bisphosphatase 1 (FBP1), SDC4, cadherin-related family member 5 (CDHR5), adenosylhomocysteinase (AHCY), and annexin A11 (ANXA11). No changes were detected after fulvestrant treatment of LILRA5, angiopoietin-related protein 7 (ANGPTL7), adhesion G-protein coupled receptor G2 (ADGRG2), CTSH.

Protein S100-P (S100P) was up-regulated in premenopausal breast tissue but failed to show any correlation with local breast E2 levels. In the murine tumors S100P levels were unaffected by fulvestrant treatment, **Figure 8B**.

Of the proteins that correlated negatively with estradiol in human tissue *in vivo* up-regulations by fulvestrant were detected for ectonucleotide pyrophosphatase/phosphodiesterase family member 7 (ENPP7), aromatic-L-amino-acid decarboxylase (DDC), dihydropteridine reductase (QDPR), integrin beta-7 (ITGB7), and tyrosine-protein kinase receptor TYRO3TYRO3, **Figure 8C**. Thus, of the 18 detectable proteins in the murine samples, 14 corroborated the results from human breast tissue.

## 4 Discussion

Here we quantified *in situ* levels of a panel of 92 key proteins for metabolism, in estrogen receptor positive (ER+) breast cancer in women, in postmenopausal women with dense or nondense breast tissue and in premenopausal women. Our data suggest that dense breast tissue expresses a similar pattern of metabolic proteins as human breast cancer whereas estradiol induces a distinctly different pattern of these proteins in the breast. An estrogen dependent regulation of several of these proteins was corroborated in a murine model of ER+ breast cancer. Thus, preventive measures against breast cancer may require different approaches depending on the risk factor.

Metabolic adaptation is one major hallmark in cancer. In addition to energy metabolism, several other metabolic pathways are affected. Understanding these tissue-specific metabolic phenotypes is fundamental for the discovery of novel therapeutic targets. How proteins in the extracellular space involved in metabolism are affected in human breast cancer is less studied. Here we show that several of these proteins are indeed up-regulated in human ER+ breast cancer *in vivo*. Additionally, our data showed that dense normal breast tissue exhibits similar expression pattern of these proteins as those found in breast cancer. Contrary, a distinct different pattern of affected proteins was shown to be estrogen dependent in normal human breast tissue.

Our data shows that the proteins with the highest fold change in breast cancer were carbonic anhydrase 13 (CA13), sulfatase-modifying factor 2 (SUMF2), and synaptosomal-associated protein 23 (SNAP23). All of these three proteins are involved in three entirely different pathways of primary metabolism; CA13, a carbonic anhydrase, contributes to cell respiration, lipogenesis and gluconeogenesis; SUMF2 inhibits the activity of SUMF1, which in turn is involved in cysteine conversions and modulations of cell metabolism; and SNAP23 a protein which contributes to membrane transport and vesicle trafficking important for insulin activities (35–37). These three proteins were also up-regulated in dense breast tissue with positive correlations with local LTF in postmenopausal women. In contrast, no changes in the regulation of these three proteins were detected in premenopausal breasts as compared to postmenopausal breast and no correlations to estradiol were revealed. The roles of these proteins in cancer progression remains elusive, but our data nevertheless suggest that they may have a role for breast cancer progression *per se* and possibly also in early stages of cancer development in dense breast tissue.

Of the 92 quantified proteins only two shared similar up-regulations in breast cancer, dense breast tissue, and in premenopausal breasts namely CTSH and GAL. CTSH is a lysosomal cysteine protease that also has been detected in secretory vesicles (38). Lysosomal and extracellular cathepsins are important for protein degradation and control of nutrient sensing and metabolic homeostasis (39). Cathepsins have also a vital role in controlling energy metabolism including the processing of lipoproteins (40–42). The role of cathepsins, including CTSH, in cancer progression is somewhat contradictory. High serum levels of CTSH in lung- and thyroid cancer have been associated with good prognosis whereas high blood and tissue levels in lung- and gall bladder cancer are associated with decreased survival (43–46). In prostate cancer CTSH has been shown to increase metastases *via* modulations of integrin activation (47). The data of CTSH in breast cancer is sparse, but our results indeed show that this cathepsin may play a role in human breast cancer.

GAL was also up-regulated in all three different settings: ER+ breast cancer, postmenopausal dense breasts, and premenopausal breasts. GAL’s role in metabolism is diverse including glucose uptake, insulin sensitivity, and growth hormone release (48). GAL expression alongside its receptors have been detected in several cancer forms including breast cancer and both pro- and anti-tumorigenic actions have been suggested (49). Previous studies have shown an estrogen dependent regulation of GAL (50). Our data support such regulation both in normal breast tissue and ER+ breast cancer as a significant positive correlation between GAL and estradiol was detected normal human breast tissue and a significantly decreased levels were found after fulvestrant therapy in experimental ER+ breast cancer in mice. Interestingly, CTSH in extracellular vesicles has been shown to be important for GAL production in brain cortex of mice (38). Our data suggest that this may indeed be the case also in human breast tissue as these two proteins correlated significantly *in vivo*.

There were 37 proteins that were significantly up-regulated in dense breast as compared to nondense breast. Of these 37, 32 correlated significantly with the continuous density measure LTF. Disabled homolog 2 (DAB2) and regenerating islet-derived protein 4 (REG4) were two proteins that were strongly associated with breast density. DAB2 is a multifunctional protein involved in many signaling pathways regulating homeostasis in cells including lipoprotein receptor regulations (51, 52). It has been suggested to be tumor suppressor but its effects on immune regulation may also indicate pro-tumorigenic effects (51).

DAB2 has also been shown to increase skin fibrosis in mice, which is in line with our results of increased levels in dense breasts, which contain elevated levels of collagen (53). REG4 is another multifunctional protein involved in cell cycle regulation, glycolytic metabolism, and diabetes (54, 55). REG4 up-regulation has been associated with cancer in the GI-tract including pancreas where it stimulates proliferation and inhibits apotosis (55). Low levels have been detected in breast cancer, which is corroborated by our data as no increase was detected in ER+ breast cancers. However, REG4 was three times higher in dense breasts as compared to nondense breast, indeed suggesting a role in normal breast physiology and possibly in breast cancer initiation and progression.

Estrogen exposure play an essential role in the control of metabolism throughout the body by affecting everything from food intake, fat cell function and distribution, peripheral insulin sensitivity and β-cell function in the pancreas, to lipid metabolism locally in blood vessels and in the liver (56). In the breast estrogen also affects the proliferation, which is related to metabolism. Even though there are limited data on hormonal regulations of the metabolic proteins analyzed in the present study it was expected that several of the proteins would correlate to estradiol. In premenopausal breast, 27 out of 28 proteins that were up-regulated as compared to postmenopausal breast correlated significantly with E2 levels. Of these, a strong negative correlation was found for ANXA4 whereas LILRA5 and SIGLEC7 were positively associated with E2. ANXA4 has an important function in membrane permeability and membrane trafficking (57). AXA4 is a negative regulator for adenylate cyclase type 5, which in turn is important for insulin secretion and cAMP production (58). Blood levels of ANXA4 have been shown to be increased in hepatocellular cancer and up-regulations in tumor tissue has been detected in colorectal- and ovarian cancer suggesting a tumor promoting effect of this protein (57). Studies of ANXA4 in breast cancer are sparse. In human endometrium a progesterone dependent up-regulation of ANXA4 has been revealed whereas estradiol did not change its levels (59). Surprisingly, our data indicates that estradiol down-regulates ANXA4 in normal human breast tissue. This suggests that ANXA4 may not be associated with estrogen dependent initiation of breast cancer. As ANXA4 was undetectable in murine tumors we could not confirm the human data. Further studies of its role in breast cancer are, however, warranted.

LILRA5, which was positively correlated with estradiol, may be important for the regulation of innate immune response as it is expressed on neutrophils. There are, however, no comprehensive analyses of its physiological function including metabolism, possible role in cancer or whether estradiol may be involved in its regulation (60). Our data suggests that estradiol may play a role in the regulation of this protein in normal breast, but this was not corroborated in ER+ breast cancer in mice. Further studies are warranted to conclude the role of estradiol in the regulation of LILRA5. The other protein that showed a strong positive correlation with estradiol was SIGLEC7. This protein may affect metabolism by interfering with the glycosylation pattern of proteins and lipids, which are important for normal cell homeostasis and cell turnover (61). In cancer, SIGLEC7 is expressed on most immune cells and can favor immune evasion in cancer, in addition to its contribution to tumor growth and progression (61). Regarding regulation of SIGLEC7 by estradiol little is known. Genomic data from rat brain has suggested a two-fold increased expression of SIGLEC1 by estradiol exposure suggesting that this protein family may be under hormonal control, which are in line with our data from breast tissue (62). SIGLEC7 was undetectable in the murine tumors.

Of the proteins that correlated with estradiol in human breast tissue, 18 were detectable in the experimental set up. Of these 18, 14 corroborated the human results suggesting that estradiol may indeed be a player of the regulation of metabolism locally in breast tissue. One limitation of the murine data is that the model is immune deficient.

The microenvironment is a rich milieu, a tissue ecosystem, in which all cells contribute to the total repertoire of proteins regulating the inter-cellular crosstalk (16, 63, 64). This intercellular crosstalk includes extracellular soluble proteins that may not be distinguished by standard molecular techniques for whole tissues such as biopsies. Microdialysis enables *in vivo* sampling of extracellular molecules that mirrors this crosstalk directly from the tissue of interest unlike blood samples that reflect secreted proteins from all different organs combined. Microdialysis is therefore a useful tool for explorative clinical studies elucidating normal physiology or pathology for the discovery of novel targets that could be further investigated. The invasiveness of microdialysis is comparable to a core biopsy, and sampling is time consuming. Due to this, the technique is unsuitable for clinical use in healthy women, such as for screening purposes.

During the progression from premalignant lesions to locally invasive cancers, intrinsic cancer cell alterations alongside microenvironmental cues may induce metabolic changes that enable cancer progression. Interactions between cancer cells with the surrounding cells in the microenvironment shape the metabolic milieu that can affect cancer progression (14). Previously metabolic reprogramming has mostly been associated with aberrant glycolysis, the Warburg effect. However, as recently reviewed, metabolic reprogramming in cancer is a complex biological trait that includes many different pathways that evolve into different metabolic phenotypes during cancer progression (15). Understanding this complex reprogramming of metabolism is necessary for the discovery of actionable therapeutic targets.

We conclude that, out of 92 proteins related to metabolism, 29 were significantly altered in human breast cancer *in vivo*. In normal breast tissue, breast density and estradiol induced two distinct different patterns of these metabolic proteins. Surprisingly, tissue density seems to be more important than estradiol for the local control of the proteins as 37 proteins were associated with density whereas only 27 were associated with estradiol. Our data, which need to be confirmed in larger cohorts of patients, suggest that metabolic proteins may indeed represent targets that warrant further for treatment and prevention of breast cancer. However, preventive measures against breast cancer may require different approaches depending on risk factor.

## Data availability statement

The original contributions presented in the study are included in the article/**Supplementary Material**. Further inquiries can be directed to the corresponding author.

## Ethics statement

The studies involving human participants were reviewed and approved by The Regional Ethical Review Board of Linköping. The patients/participants provided their written informed consent to participate in this study. The animal study was reviewed and approved by The Institutional Animal Ethics Committee at Linköping University.

## Author contributions

CD designed the project and performed all microdialysis investigations and the animal study. AA participated in acquiring the microdialysis samples, carried out sample preparation, and participated in the animal study. PL design and analyzed the MRI part of the study. CD, JE, and PL analyzed the data and prepared the manuscript. All authors contributed to the article and approved the submitted version.
