# Supplementary figures and images for: Breast density and estradiol are associated with distinct different expression patterns of metabolic proteins in normal human breast tissue in vivo
Source: Front Oncol. 2023 Mar 29;13:1128318. doi: 10.3389/fonc.2023.1128318 (PMC10090464; doi:10.3389/fonc.2023.1128318)

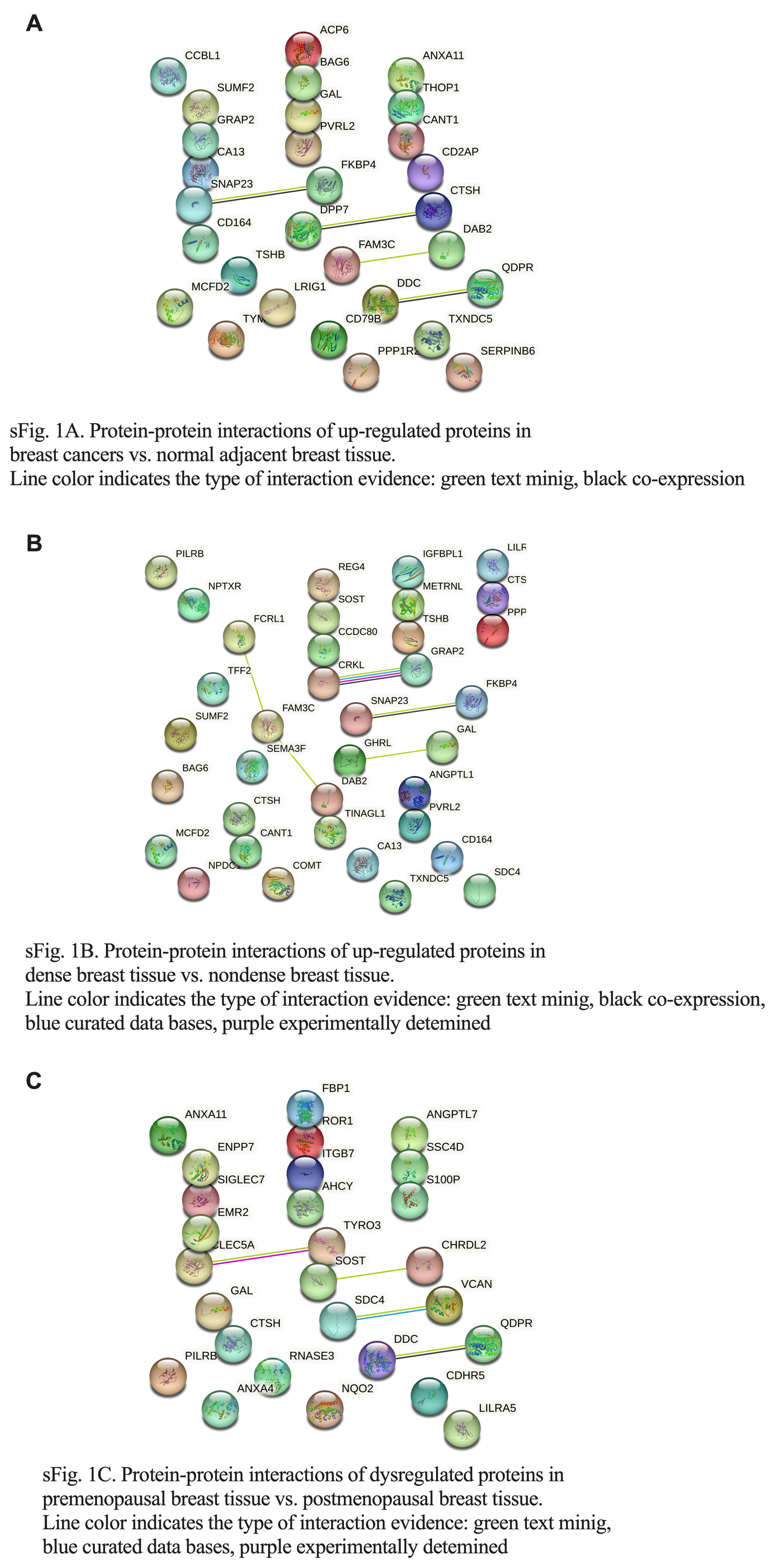

Supplement: Supplementary file 1 [file Image_1.tiff]
